# Cultivating the unseen: Lessons from James Tiedje

**DOI:** 10.1002/mlf2.12083

**Published:** 2023-09-24

**Authors:** Yoichi Kamagata

**Affiliations:** ^1^ National Institute of Advanced and Industrial Science and Technology (AIST) Tsukuba Japan

In recounting Dr. James M. Tiedje's outstanding research achievements spanning the past 55 years, it is easy to overlook his early and mid‐career endeavors. Specifically, his contribution to the aerobic degradation of pesticides and other chemicals, as well as methanogenic degradation of those compounds retains their brilliance. Many researchers in environmental microbiology have gained invaluable knowledge from these studies, which have been applied to the elucidation of previously uncultivated microorganisms.

Dr. Tiedje embarked on his career in soil microbiology at Cornell University in 1964 under the guidance of Martin Alexander. Motivated by Rachel Carson's Silent Spring published in 1962, he developed a keen interest in studying the degradation of 2,4‐dichlorophenoxy acetic acid (2,4‐D), widely used as a broad‐leaf herbicide. Dr. Tiedje found that an *Arthrobacter* species converts 2,4‐D into chlorocatechols, facilitated by a soluble ether linkage‐cleaving enzyme^1^, ^2^. Subsequently, extensive investigations into the 2,4‐D degradation by aerobic microorganisms were conducted, leading to the identification of α‐ketoglutarate‐dependent dioxygenase, the enzyme involved in the first step of 2,4‐D metabolism^3^ (Figure 1). The story starts with my involvement in the “2,4‐D project.”

**Figure 1:**
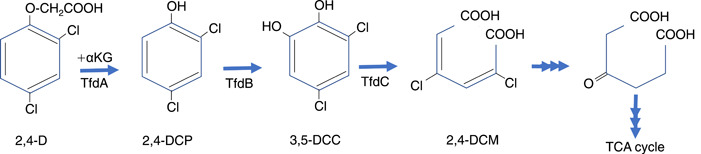
Proposed pathway for 2,4‐D degradation in an aerobic bacterium *Cupriavidus pinatubonensis* (formerly *Alcaligenes eutrophus*). 2,4‐D, 2,4‐dichlorophenoxy acetic acid; 2,4‐DCM, 2,4‐dichloromuconate; 2,4‐DCP, 2,4‐dichlorophenol; 3,5‐DCP, 3,5‐dichlorocatechol; αKG, α‐ketoglutarate. TCA, tricarboxylic acid cycle.

## MICROBIAL DEGRADATION OF 2,4‐D

The project took place at the Center for Microbial Ecology, Michigan State University (MSU), where we focused on microbial evolution. 2,4‐D, being an anthropogenic chemical with no analogous compounds found in nature, provided an excellent opportunity to explore how enzymes with different original functions were recruited and evolved to adapt to 2,4‐D degradation. The project was initiated by Profs. James M. Tiedje and Keiji Yano (followed by Prof. Koki Horikoshi) in 1991 and received funding from Japan Science and Technology Agency (JST, formerly JRDC) and National Science Foundation USA. It involved numerous scientists and over 10 postdocs from various parts of the world. It was the mid‐1990s, a time before high‐throughput genome sequencing became available, and molecular biological studies were conducted using classical methods such as DNA sequencing using big gel plates. During this period, I had been working on methanogenic Archaea till I joined the project.

To begin, we initiated genetic analysis of known 2,4‐D‐degraders, as well as search for previously unknown 2,4‐D‐degrading microbes^4^, ^5^. Meanwhile, Dr. Tiedje, who was supposed to lead the project, was on sabbatical, enjoying the warmer climate of Hawaii (quite different from Michigan) and collaborating with Hawaiian researchers. He learned that there were soils in Hawaii that had not been exposed to 2,4‐D, and he brought those untouched soils back to Michigan. The underlying idea was to investigate whether the microorganisms capable of breaking down this widely used chemical, which was artificially synthesized and sprayed in large quantities, were absent in the Hawaiian soils, or there were microbes possessing the prototypic enzyme. I inoculated a liquid culture containing 2,4‐D with Hawaiian soil and spent day after day monitoring its degradation by liquid chromatography. Our initial expectation was that 2,4‐D decomposition would not occur so readily.

Indeed, for a while, no 2,4‐D degradation was observed. However, after a few weeks or perhaps a month, some soil samples began to show signs of degradation. I was secretly excited that the 2,4‐D‐degrading microorganisms might be present in these remote regions that had never been associated with 2,4‐D. Are the 2,4‐D‐degrading microorganisms entirely novel or are they the same *Alcaligenes* (currently called *Curpriavidus*) species that Dr. Tiedje and his team as well as everyone here at MSU have been studying for years?

However, it was not easy to clarify the true nature of the microorganisms that had emerged in the test tube. This was primarily due to their inability to readily form colonies on agar media. Although numerous colonies appeared on the agar medium containing 2,4‐D, it was evident that this was a result of adding more yeast extract than 2,4‐D. Moreover, it was also clear that 2,4‐D was not degraded in liquid culture without yeast extract. In addition, the Hewlett‐Packard liquid chromatograph frequently malfunctioned, causing delays in analysis and hindering our ability to identify the problem. To overcome these challenges, we decided to conduct an experiment in which we added radioactive ^14^C‐labeled 2,4‐D to visualize which colonies would show the highest concentration of radioactivity.

Numerous microorganisms grew in a myriad of locations on the agar medium containing 2,4‐D. Radioactive colonies were scattered among them in a hidden manner. It was obvious that the only way to eliminate microorganisms unrelated to 2,4‐D degradation was to significantly reduce the concentration of yeast extract, which would also slow down the growth of 2,4‐D degraders. After months of intensive culture and isolation operations, we successfully isolated five 2,4‐D‐degrading microorganisms. Surprisingly, these strains were not the known 2,4‐D‐degrading bacteria, but rather closely related to *Bradyrhizobium*, a soil‐dwelling microbe that is ubiquitous^6^. Five years later, one of the isolates (obtained from soil covered for 4800 years beneath a lava flow, effectively isolated from human impact before the colonization of Hawaii) was found to harbor an aromatic ring‐hydroxylation dioxygenase (i.e., Rieske nonheme iron oxygenase), which is quite different from previously known 2,4‐D metabolic enzymes^7^, ^8^. Since then, this enzyme and its relatives have been discovered to be widely distributed among soil microorganisms. However, the evolutionary origin of this enzyme still remains enigmatic.

## THE DAWN OF HALORESPIRATION AND SYNTROPHY STUDIES

Before my involvement in the 2,4‐D project, my focus was on cultivating methanogenic archaea (methanogens) and relevant microorganisms to elucidate the functions of key microorganisms in methane fermentation processes. I encountered challenges in culturing these organisms. However, working on the 2,4‐D project reminded me that the difficulty of isolating microorganisms extends beyond anaerobic microbes and is a universal challenge in microbiology. It highlighted the crucial importance of isolation and cultivation in obtaining knowledge about their functions and characteristics. During that time, massive sequencing and metagenomic analyses had not yet emerged. We were far from a time when we could gain insights from genome sequences.

**Figure 2:**
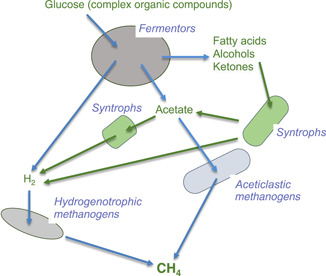
Anaerobic metabolisms of organic compounds. Organic compounds, for example, glucose can be metabolized by at least four trophic groups (including syntrophs) in the methanogenic community.

After concluding my research on 2,4‐D, I redirected my attention to syntrophs and methanogens under anaerobic conditions, a field that had long captivated my interest. Methanogens produce methane from very limited low‐molecule compounds, specifically, hydrogen + CO_2_, formate, acetate, alcohols, and methyl compounds. These substrates for methanogenesis are derived from fermentative microbes that utilize high‐molecule organic matters^9^ (Figure 2). Among them, some organisms, commonly referred to as syntrophs, are specifically associated with methanogens. Syntrophs break down (i.e., anaerobically oxidize) fatty acids, alcohols, and aromatic compounds, which are extremely difficult to be degraded for most of anaerobic microbes that utilize higher‐molecule organic compounds, and they produce hydrogen and acetate in the process. The accumulation of hydrogen and acetate is thermodynamically unfavorable, and syntrophs cannot survive unless such products are immediately eliminated from the system or metabolized by other microorganisms. Methanogens are making a major contribution as scavengers of hydrogen and acetate.

While research on syntrophs had been in its infancy for a long time^9^, ^10^, it was in the 1980s that research into their entities really began to take off. The researchers leading the way in this research field from its inception were Marvin Bryant and his group at the University of Illinois at Urbana‐Champaign^11^, ^12^, ^13^, ^14^. Dr. Tiedje also embarked on important research on syntrophs during this period. In the early 1980s—over 40 years ago, he obtained a simplified microbial population that degraded 3‐chlorobenzoate to produce methane^15^, ^16^, ^17^. The discovery of anaerobic degradation of halogenated aromatic compounds was groundbreaking at the time. That is why a short but important paper was published in Science in 1982^15^. Dr. Tiedje and his colleagues made the initial discovery of how a model halogenated compound, 3‐chlorobenzoate, could be converted into methane. They began thoroughly studying the microorganisms in the consortium. This was accomplished by culture, observation, and chemical analysis but no genetics at all as there was no genome information on the Earth^16^, ^17^, ^18^, ^19^, ^20^, ^21^, ^22^, ^23^, ^24^. The 3‐chlorobenzoate study reached a significant milestone with the complete isolation of strain DCB‐1, which was later named *Desulfomonile tiedjei* ^21^. Until then, no one had ever imagined that anaerobic microorganisms can conserve energy by using halogenated compounds as the terminal electron acceptor. Eventually, Dr. Tiedje's team (including Joseph Suflita, Daniel Shelton, Jan Dolfing, William Mohn, and others) revealed that 3‐chlorobenzoate is dechlorinated by reductive dehalogenation to benzoate, which is broken down into acetate and H_2_/CO_2_ by a syntrophic bacterium, and the H_2_/CO_2_ is converted into methane by a methanogenic archaeon, while the acetate is used as an electron donor for dechlorination^25^, ^26^, ^27^, ^28^. The series of studies showed that anaerobic syntrophy is not only involved in degradation of simple fatty acids but also more complex compounds, for example, halogenated aromatic compounds.

## CULTIVATING THE UNSEEN MICROBES

Returning to our research, the fact that most of the anaerobic microorganisms were yet to be cultivated and that syntrophic metabolism in methanogenic environments remained largely unknown was a major motivating factor for our research. In the late 1990s, microbial ecology had made significant advancements through the utilization of 16S ribosomal RNA/DNA sequencing and fluorescence in situ hybridization (FISH) analysis, thanks to the pioneering work of Amann et al., which became powerful tools^29^. In 1995–1997, the complete genomes of *Haemophilus influenzae*, *Bacillus subtilis, Synechocystis* sp., and *Escherichia coli* were sequenced, but it was still some time before the era of high‐throughput genome/metagenome sequencing would emerge.

We first attempted to isolate and cultivate syntrophs that degrade acetate, propionate, and butyrate under thermophilic conditions. It was known that methane fermentation at around 50°C was quite effective for degradation of a variety of chemical compounds. However, the isolation process proved to be quite challenging. Even when a stable enrichment culture could be obtained, it was not easy to isolate the protagonist even if FISH can identify them.

A potential solution to this problem was already hinted at early studies of syntrophs, where they were found to be capable of growing on their own without a partner organism, given certain substrates, such as *D. tiedjei* in pure culture with pyruvate and rumen fluid^20^, or *Syntrophomonas wolfei* in pure culture with crotonate^30^. Indeed, this method sometimes worked. We were eventually able to isolate a wide variety of anaerobic syntrophic microorganisms and their partner organisms (mainly methanogens) over the past 20 years, as summarized in Table 1, which includes corresponding studies^31^, ^32^, ^34^, ^35^, ^36^, ^37^, ^38^, ^39^, ^40^, ^41^, ^42^, ^43^, ^44^, ^45^, ^46^, ^47^, ^48^, ^49^, ^50^, ^51^, ^52^, ^53^, ^54^, ^55^, ^56^, ^57^, ^58^, ^59^, ^60^, ^61^, ^62^, ^63^, ^64^, ^65^, ^66^, ^67^, ^68^, ^69^, ^70^, ^71^, ^72^. It is important to note that most of these microbes are fastidious or recalcitrant, and took a long time to be isolated and cultured. Fortunately, these microorganisms have now gained widespread recognition among researchers, and their names are commonly referenced in a number of metagenomics articles.

**Table 1:** List of representative isolates that have been yet to be cultured including syntrophic bacteria and methanogenic archaea in our studies.

Name	References			Characteristics
*Thermacetogenium phaeum*	[31, 32, 33]			Acetate‐oxidizing H_2_‐producing syntroph. Grows syntrophically on acetate but grows acetogenically on methanol and some substrates in pure culture.
*Pelotomaculum thermopropionicum*	[34, 35, 36]			Propionate‐oxidizing H_2_‐producing syntroph. Grows syntrophically on propionate but grows in pure culture on pyruvate and fumarate.
*Syntrophothermus lipocalidus*	[37]			Butyrate‐oxidizing H_2_‐producing syntroph. Grows syntrophically on long‐chain fatty acids but grows in pure culture on crotonate.
*Pelotomaculum terephthalicum*	[38, 39]			Terephthalate‐oxidizing H_2_‐producing syntroph. Grows syntrophically on phthalate isomers but grows in pure culture on crotonate, hydroquinone, and hydroxybenzoate. Grows slowly.
*Pelotomaculum isophthalicum*	[38, 39]			Isophthalate‐oxidizing H_2_‐producing syntroph. Grows syntrophically on phthalate isomers and benzoate. Can be maintained only in a defined coculture. Grows slowly.
*Syntrophorhabdus aromaticivorans*	[40, 41, 42]			Phenol‐oxidizing H_2_‐producing syntroph. Grows syntrophically on phenol, *p*‐cresol, isophthalate, and benzoate. Can be maintained only in a defined coculture. Grows slowly.
*Tepidanaerobacter syntrophicus*	[43]			Ferments carbohydrates in pure culture. Grows syntrophically on lactate and ethanol.
*Anaerolinea thermophila*	[43, 44]			Carbohydrate‐utilizing bacterium. Grows better syntrophically with methanogens.
*Anaerolinea thermolimosa*	[45]			Carbohydrate‐utilizing bacterium. Grows better syntrophically with methanogens.
*Levilinea saccharolytica*	[45]			Carbohydrate‐utilizing bacterium. Mesophilic. Grows slowly.
*Leptolinea tardivitalis*	[45]			Carbohydrate‐utilizing bacterium. Mesophilic. Grows slowly.
*Bellilinea caldifistulae*	[46]			Carbohydrate‐utilizing bacterium. Grows slowly but grows better syntrophically with methanogens.
*Longilinea arvoryzae*	[46]			Carbohydrate‐utilizing bacterium. Grows slowly but grows better syntrophically with methanogens.
*Atribacter laminatus*	[47]			Carbohydrate‐utilizing bacterium. Grows better syntrophically with methanogens. First isolate within the phylum *Atribacterota*. Harbors three unique lipid layers.
*Prometheoarchaeum syntrophicum*	[48]			Asgard archaeon related to *Lokiarchaeota* from deep marine sediment. One of the most ancient archaea that might have been involved in eukaryogenesis. Grows extremely slowly but does better with hydrogenotrophic methanogens.
*Methanothermobacter tenebrarum*	[49]			Thermophilic hydrogenotrophic methanogen. It is a close relative to *Methanothermobacter thermautotrophicus* ^50^, but it was very difficult to isolate as it tends to associate with partner organisms.
*Methanocella paludicola*	[51]			Mesophilic hydrogenotrophic methanogen. It was the first isolate within the “Rice Cluster 1” that is abundant in rice paddy fields. Grows slowly.
*Methanolinea tarda*	[52]			Moderately thermophilic hydrogenotrophic methanogen. Grows slowly.
*Methanothrix pelagica*	[53]			Mesophilic aceticlastic methanogen isolated from sea water. Grows slowly.
*Methanocalculus pumilus*	[54]			Mesophilic hydrogenotrophic methanogen isolated from marine sediment. Grows slowly.
*Methermicoccus shengliensis*	[47, 55, 56]			Mesophilic methoxydotrophic methanogen. Originally isolated from a subsurface environment by Cheng et al.^56^ and another strain was later isolated in our group. Both strains can utilize a wide range of methoxylated aromatic compounds as energy sources, for which we proposed methoxydotrophy, a novel metabolism in methanogens.
*Methanolobus profundi*	[57]			Mesophilic methylotrophic methanogen isolated from a deep subsurface sediment in a natural gas field.
*Methanohalophilus levihalophilus*	[58]			Mesophilic methylotrophic, slightly halophilic methanogen isolated from a deep subsurface sediment in a natural gas field.
*Methanomicrobium antiquum*	[59]			Mesophilic hydrogenotrophic, slightly halophilic methanogen isolated from a deep subsurface sediment in a natural gas field.

*Thermoacetogenium phaeum*, an acetate‐oxidizing and hydrogen‐producing microbe, was the first syntroph that the author's group isolated^31^. We learnt a lot from this bacterium about how syntroph behaves and makes a living^31^, ^32^, ^60^, ^61^, ^62^. It can switch between acetogenesis and reverse acetogenesis under different conditions. The latter (i.e., syntrophic growth) was only observed when hydrogenotrophic methanogen exists as an H_2_ consumer. This microbe was also found to drive the Wood–Ljungdahl pathway in both directions^62^. As for acetate metabolism, it had been widely believed that aceticlastic (acetotrophic) methanogens play a primary role in methanogenic environments. However, many reports indicate the importance of syntrophic acetate oxidation coupled with H_2_‐consuming methanogenesis. Shigematsu et al. investigated acetate conversion pathways of methanogenic consortia in acetate‐fed chemostats at dilution rates of 0.025 and 0.6 day^−1^ and found that nonaceticlastic syntrophic oxidation by acetate‐oxidizing syntrophs and hydrogenotrophic methanogens dominated over aceticlastic methanogens at the low dilution rate, whereas aceticlastic cleavage was suggested to occupy a primary pathway in total methanogenesis at the high dilution rate^73^. We found that deep subsurface environments harbor both thermophilic acetate‐oxidizing syntrophs and aceticlastic methanogens. Furthermore, the population changes depending on CO_2_ concentrations^33^. Due to the limited availability of acetate‐oxidizing syntroph strains, our isolate has become a valuable model organism to study the population dynamics over acetate metabolism.

Phenol had long been known to be degraded under methanogenic conditions, but the microbes responsible for the degradation reaction were not known at all. At least, it was evident that phenol can be only syntrophically metabolized and it is very challenging to reveal the entity of the microbes. *Syntrophorhabdus aromaticivorans* was the first tangible, obligately anaerobic, syntrophic organism capable of oxidizing phenol in association with a H_2_‐scavenging methanogen partner^40^, ^41^, ^42^. It could metabolize not only phenol but also *p*‐cresol, 4‐hydroxybenzoate, isophthalate, and benzoate. Since numerous reports based on metagenomic analyses indicate that *Syntrophorhabdus* type of bacteria is abundant in phenol‐degrading methanogenic communities and there are no other possible microbes (except for denitrifying phenol degraders), the organism may monopolize phenol degradation. The whole‐genome analysis showed that *S. aromaticivorans* syntrophic phenol‐degrading phenylphosphate synthase (PpsAB) and phenylphosphate carboxylase (PpcABCD) catalyze the first two steps of phenol metabolism into benzoate. It also shows benzoate degradation through hydration of the dienoyl‐coenzyme A (CoA) intermediate as reported in *Syntrophus aciditrophicus*. The conversion of benzoyl‐CoA into dienoyl‐CoA is an extremely endergonic reduction; thus, it may be catalyzed by electron‐bifurcating reduction that involves benzoyl‐CoA reductase, hydrogenase, and heterodisulfide reductase.

Studies on syntrophy culminated in the discovery and isolation of archaeon whose ancestor might have been the closest relative to eukaryotes^48^. Imachi et al. took deep‐sea methane‐seep sediment samples and enriched microbes by a continuous down‐flow reactor using sponge as a carrier. After 12 years of dedicated efforts and various attempts, the microbe was isolated in coculture with *Methanogenium* sp. The archaeon named *Candidatus Prometheoarchaeum syntrophicum* is an anaerobic, extremely slow‐growing, small coccus capable of metabolizing amino acids through syntrophy. The reason that the isolate organism is referred to as “candidatus” is simply because it has not yet been deposited to public culture collections due to the difficulty in yielding sufficient amounts of biomass to maintain and distribute. The microbe was the first cultured Asgard archaeon, harboring 80 eukaryotic‐like proteins, and has characteristic protrusions. Imachi et al. proposed an attractive hypothesis for eukaryogenesis, in which a primordial Asgard archaeon cell interacts with the ancestral beneficial bacterial cells and eventually endogenizes it, which is termed the entangle–engulf–endogenize (E3) model. The model has recently been reinforced by the discovery of another archaeon named *Candidatus Lokiarchaeum ossiferum* enriched from sediments from a small estuarine canal^74^.

## CONCLUDING REMARKS

From the 1970s to the 1990s, microbiology focused on cultivation, biochemistry, physiology, and genetics. Dr. Tiedje was one of the greatest contributors to this era. With the arrival of the 21st century and the emergence of massive metagenome data, microbiology, and environmental microbiology have transitioned to a new stage. Despite this shift, most of the results obtained from this stage have been a continuation of previous research. It should be noted that microbiology still heavily relies on the process of isolation and cultivation to uncover the truth behind morphology, physiology, metabolism, and energetics. Data science will reinforce the future of microbiology.

Dr. Tiedje, on the other hand, was one of the first researchers to recognize the importance of data science. In 1997, he and Jim Cole took over ribosomal database project (RDP) that had been run by Profs. Carl Woese, Gary Olsen, and colleagues at the University of Illinois Urbana‐Champaign^75^. The RDP had 1687 aligned sequences (now millions!) and all sequences were curated by Carl Woese himself in 1993 when Dr. Tiedje made the decision to take it over. Today, microbial ecology heavily relies on this database and relevant ones. In addition, Dr. Tiedje has dedicated himself to studying global‐scale microbiomes including wastewater treatment plants, rain forests, permafrost, nitrogen‐fertilized soils, and antibiotic‐contaminated environments, all within the context of human issues. He consistently remains at the forefront of his field. I would like to express my utmost respect and always remember his earlier studies, which tends to be overlooked by many.
